# Pancreatic ascites managed with a conservative approach: a case report

**DOI:** 10.1186/s13256-020-02463-0

**Published:** 2020-09-15

**Authors:** Raju Bhandari, Rajan Chamlagain, Saraswati Bhattarai, Eric H. Tischler, Rajesh Mandal, Ramesh Singh Bhandari

**Affiliations:** 1 Department of General and GI Surgery, Tribhuvan University Teaching Hospital, Kathmandu, Nepal; 2 Tribhuvan University, Institute of Medicine, Maharajgunj, Kathmandu, Nepal; 3 Department of Orthopedic Surgery and Rehabilitation Medicine, State University of New York, Downstate Medical Center, Brooklyn, New York, USA

**Keywords:** Pancreatitis, Ascites, Surgical, Medical, Case report

## Abstract

**Background:**

Pancreatic ascites refers to the massive accumulation of pancreatic fluid in the peritoneal cavity and is a rare entity. Chronic alcoholic pancreatitis is the most common cause. Ascites is commonly seen in patients with alcoholic liver disease and is usually a consequence of portal hypertension. Biliary pancreatitis, pancreatic trauma and cystic duplications of biliopancreatic ducts, ampullary stenosis, or ductal lithiasis are the remaining causes.

**Case presentation:**

A 53-year-old Chhetri man, a chronic alcoholic, presented with epigastric pain and abdominal distension. He had made several previous visits to a local hospital within the past 6 months for a similar presentation. Serum alkaline phosphatase 248 IU/L, serum amylase 1301 IU/L, and lipase 1311 IU/L were elevated while serum calcium was decreased (1.5 mmol/l). Ascitic fluid amylase was elevated (2801 IU/L). A computed tomography scan of his abdomen revealed features suggestive of acute-on-chronic pancreatitis. The case was managed with a conservative approach withholding oral feedings, starting total parenteral nutrition, paracentesis, octreotide, and pigtail drainage.

**Conclusion:**

Pancreatic ascites is a rare entity. Diagnosis is suspected with raised ascitic fluid amylase in the presence of pancreatic disease. Such cases can be managed by conservative approach or interventional approach. We managed this case through a conservative approach.

## Background

Massive ascites in a chronic alcoholic patient is usually attributed to hepatic cirrhosis [1]. Pancreatic ascites should be suspected in patients with chronic alcoholism and pancreatitis presenting with ascites [2]. The etiology is probably a pancreatic pseudocyst leakage or ductal disruption [3]. The diagnosis is based on demonstration of ascitic fluid amylase (> 1000 U/L). Chronic pancreatitis (83%), acute pancreatitis (8.6%), and trauma (3.6%) are common causes for ductal disruption. Medical treatment includes withholding oral feedings, total parenteral nutrition (TPN), paracentesis, and administering octreotide [4]. For those not responding to medical therapy, interventional therapy may be needed which includes endoscopic transpapillary pancreatic duct stenting or surgery which includes cystogastrostomy, cystenterostomy, pancreatic sphincterectomy, or partial pancreatic resection [5–7]. We present a case of massive ascites in a patient with chronic pancreatitis secondary to chronic alcohol use. The case was successfully managed with a combination of medical and interventional therapy.

## Case presentation

A 53-year-old, Chhetri man with a history of 10–12 years of chronic alcoholism presented to our hospital with the chief complaints of weight loss of 18 kg over the past 6 months, as well as epigastric pain and vomiting for the past month. Otherwise, there was no documented history of fever, yellowish discoloration of skin, gastrointestinal bleeding, melena, dark-colored urine or pale stool. His past medical or surgical history was not significant. He had been admitted to a local hospital on several occasions in the past 6 months with similar complaints and was diagnosed as having acute mild pancreatitis, which was managed conservatively. His current pain was moderate to severe in intensity, radiating to his back, and it was aggravated by meals and relieved on stooping forward. He had associated symptoms of non-projectile, non-bloody, and non-bilious vomiting, only containing water and food contents. Furthermore, he also complained of respiratory discomfort that was concurrent with pain episodes. On examination, he was ill appearing and had the following vital signs: pulse of 124 beats per minute, temperature 37 ºC (98.6 ºF), and blood pressure of 110/70 mm Hg. An abdominal examination revealed marked generalized abdominal tenderness. Bowel sounds were normal. Other systemic examinations were not significant.

With regards to laboratory values, total cell and differential counts, electrolytes, bilirubin, serum glutamic-oxaloacetic transaminase (SGOT)/serum glutamic-pyruvic transaminase (SGPT), lactate dehydrogenase (LDH), serum protein, serum albumin, and urine and stool analysis were within normal limits at the time of admission. However, serum alkaline phosphatase (ALP; 248 IU/L), amylase (1301 IU/L), and lipase (1311 IU/L) were elevated while serum calcium was decreased (1.5 mmol/l). Tumor markers CA 19-9 and carcinoembryonic antigen (CEA) were within normal limits. Arterial blood gas analysis revealed respiratory alkalosis: pH = 7.48, partial pressure of carbon dioxide (pCO_2_) 28 mmHg, and bicarbonate (HCO_3_) 21.3 mmol/l. Ultrasonography (USG) of his abdomen and pelvis revealed features suggestive of complicated acute pancreatitis with loculated peripancreatic collection extending to the bilateral perinephric space; however, it was noted to be more prominent on the left side. He was admitted with the diagnosis of acute pancreatitis and treated conservatively. He was kept nil by mouth with the initiation of TPN, octreotide infusion, intravenous fluid (Ringer’s lactate solution; RL), morphine, and paracetamol. A central venous catheter was inserted.

**Fig. 1:**
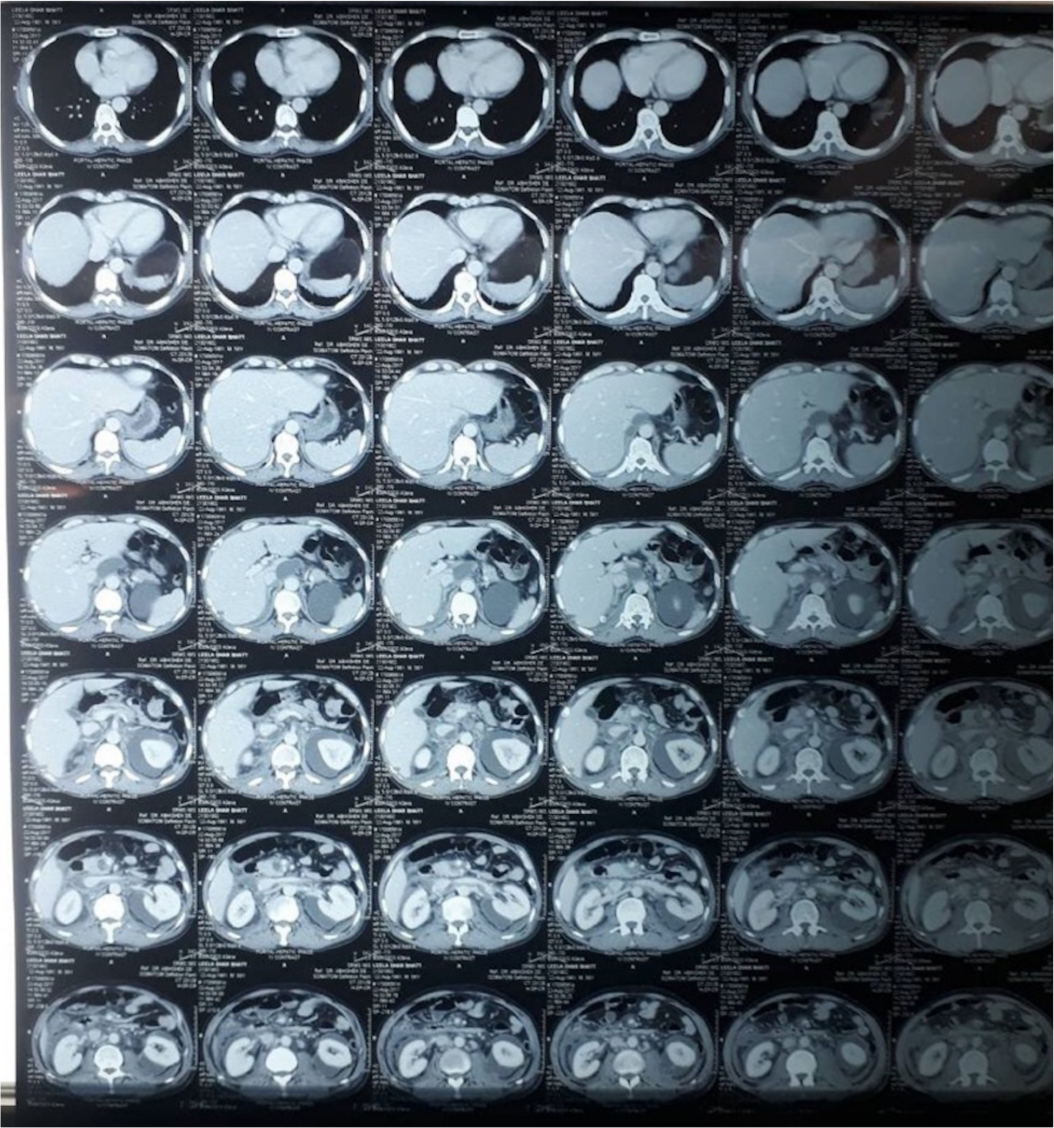
Computed tomography scan of the abdomen and pelvis showing decreased pancreatic bulk and small cystic area in uncinate process with adjacent peripancreatic and retroperitoneal collection extending to bilateral pararenal space

On the third day of admission, he developed abdominal distension with pain and maximal fever of 37.8 ºC (100.1 ºF). USG-guided diagnostic tapping of ascitic fluid was performed. Ascitic fluid analysis revealed white blood cell count of 1740 cells/mm^3^ with 60% granulocytes, total protein of 3.6 g/dl, and albumin of 1.8 g/dl. Blood culture was negative at that time. On subsequent days, leukocyte counts decreased to 13,000/mm^3^ from 17,000/mm^3^. Ascitic fluid amylase was 2801 IU/L and adenosine deaminase was 11 U/ml. Serum ascites albumin gradient (SAAG), the difference in serum and ascitic fluid albumin level, was 1.0 g/dl signifying non-portal cause. A computed tomography (CT) scan of his abdomen and pelvis revealed decreased pancreatic bulk with mildly prominent pancreatic duct and a small cystic area in the uncinate process with adjacent peripancreatic and retroperitoneal collection extending to bilateral pararenal space, which suggested acute-on-chronic pancreatitis (Fig. 1).

On the tenth day of admission, a 10F pigtail drainage was inserted in his right pelvic cavity in paracolic gutter which drained approximately 472 ml. Previously, the drainage from the same site at first day was 3000 ml and 20 ml on eighth day. Meanwhile, abdominal girth reduced from 78 cm to 68 cm during the same duration. He improved clinically and symptomatically and was discharged on the 22nd day of admission. On a follow-up clinic visit 1 week later, our patient was noted to have marked improvement in abdominal distension and discomfort.

## Discussion

Pancreatic ascites refers to the massive accumulation of pancreatic fluid in the peritoneal cavity. The prevalence rate of pancreatic ascites is only 1%; it is more common in men than in women (male:female ratio 2:1) and between 20 and 50 years of age [8]. Ascites is commonly seen in patients with alcoholic liver disease and is usually a consequence of portal hypertension. Biliary pancreatitis, pancreatic trauma and cystic duplications of biliopancreatic ducts, ampullary stenosis, or ductal lithiasis account for the remainder of cases [9]. It occurs in approximately 4% of patients with chronic pancreatitis and 6–14% of patients with pancreatic pseudocysts. It may also occur after an incidence of acute pancreatitis or blunt abdominal trauma causing duct dehiscence. The diagnosis should be considered in patients with chronic ascites with a history of alcoholism, chronic pancreatitis, or abdominal trauma [8]. In 80% of cases, pancreatic ascites results from leakage from a pseudocyst in communication with the ductal system; ductal disruption in the absence of pseudocyst accounts for the remaining 20% of cases [5].

Patients with pancreatic ascites often present with symptoms of mild abdominal pain, decreased appetite and sense of fullness, weight loss, and progressive ascites. They often present with a history of chronic pancreatitis, a recent episode of acute pancreatitis, or with new-onset ascites. However, these symptoms may be absent in alcoholics and the diagnosis may be falsely attributed to cirrhosis. Diagnosis can be made by paracentesis and analysis of the fluid for protein and amylase content. Pancreatic ascites is an exudative ascites characterized by high amylase concentration in ascitic fluid (usually over 1000 IU/L) and protein concentration over 3 g/dl, which differentiates it from cirrhosis, tuberculosis, or carcinomatosis. Rarely, the origin is indeterminate in 10% of cases. In addition to ascites, pleural effusion may be seen in such cases [10].

Once the diagnosis has been established, abdominal CT should be done to rule out pseudocysts. The use of endoscopic retrograde cholangiopancreatography (ERCP) has been suggested to help localize ductal obstruction or the site of leakage for stenting when possible. Magnetic resonance cholangiopancreatography (MRCP) can delineate the anatomy of pancreatic duct and any abnormalities present and can be considered for candidates who cannot undergo ERCP [11]. However, the role of MRCP in the diagnosis of pancreatic ascites is not clear due to a paucity of data.

Therapy for pancreatic ascites is controversial [8]. There are no randomized control studies regarding therapy due to the rarity of the condition. A patient with pancreatic ascites may be managed conservatively withholding oral feedings and starting TPN. These minimize pancreatic secretions. One third of patients usually improve with this conservative approach while some may require treatment with octreotide or other somatostatin analogs, diuretics, and repeated paracentesis. Segal *et al.* conducted a prospective study to evaluate the efficacy of a long-acting somatostatin analogue called Sandostatin (octreotide) among 18 patients with either pancreatic ascites or external pancreatic fistulas (12). The ascites resolved in nine out of ten patients in a mean period of 22 days (+/− 3 days). The external fistulas were all high-output fistulas and resolved in seven out of eight patients. These results indicate the value of conservative patient management [12].

The total duration of therapy is unknown and a trial of 4–6 weeks is suggested with a consideration for interventional therapy if the condition does not resolve. This approach led to healing in less than 50% of patients and overall mortality of 15% and 15–25% recurrence [9].

Fortunately, our patient improved with conservative management. Patients who fail conservative therapy require interventional therapy which can be either endoscopic or surgical. ERCP is a valuable tool in the evaluation of patients with pancreatic ascites to locate the site of disruption and, subsequently, placement of a transpapillary stent to bypass the obstruction in addition to large-volume paracentesis. In addition, patients can undergo concomitant endoscopic or percutaneous pseudocyst drainage [8].

Surgical therapy is recommended when there is no response to conservative therapy in 3–4 weeks. The choice of surgery depends on the site of leakage and associated pancreatic abnormality as demonstrated by ERCP and contrast-enhanced CT. Pseudocysts are usually treated by distal pancreatectomy when the leak is in the pancreatic tail or drained by cystogastrostomy, cystojejunostomy, or cystoduodenostomy. Studies have shown that internal pancreatic drainage is the ideal surgical treatment for patients with pancreatic ascites and/or pleural effusion that do not respond to medical treatment. When this is not feasible, external drainage can be used as an alternative to pancreatic resection [7]. Fistulas in the pancreatic duct are usually drained to a Roux-en-Y jejunal loop. Recurrence rates from 50 to 64% have been reported in patients undergoing surgical intervention without ERCP [8]. Mortality rates have been reported to be similar with surgical and medical therapies (15–25%) [8].

We successfully managed our patient with a conservative approach. Our case highlights a few important messages. First, we often label the ascites as cirrhotic or secondary to portal hypertension given its high prevalence; it is important to also consider pancreatic ascites as part of the differential. As a clinician, it is important not to miss cases of pancreatic ascites. Past history or recent history of pancreatitis, recurrent admissions to an emergency room due to abdominal pain in chronic alcoholics, or history of blunt abdominal trauma may provide important guidance in diagnosing such cases. Furthermore, evaluation of the SAAG can be accurately measured to narrow the diagnosis. Paré et al. reported that SAAG calculation offers the best diagnostic discrimination between ascites caused by liver disease and ascites caused by a neoplasm [13]. Second, such cases are typically rare, and if diagnosed can be managed with medical and/or interventional therapy.

## Conclusion

Alcoholic chronic pancreatitis is the most common cause of pancreatic ascites; it is a rare entity. Biliary pancreatitis, pancreatic trauma and cystic duplications of biliopancreatic ducts, ampullary stenosis, or ductal lithiasis account for the remainder of cases. Diagnosis is usually clinched by raised ascitic fluid amylase (> 3 times that of plasma), raised total ascitic protein level (> 3 g/dl) and low SAAG (< 1.1 g/dl) in the presence of pancreatic disease, and such cases can be managed by a conservative approach.

## Data Availability

All data are within the article.

## Abbreviations

TPN: Total parenteral nutrition
CEA: Carcinoembryonic antigen
CT: Computed tomography
ERCP: Endoscopic retrograde cholangiopancreatography
MRCP: Magnetic resonance cholangiopancreatography
HCO_3_: Bicarbonate
LDH: Lactate dehydrogenase
ALP: Alkaline phosphatase
pCO_2_: Partial pressure of carbon dioxide
RL: Ringer’s lactate solution
SGOT: Serum glutamic-oxaloacetic transaminase
SGPT: Serum glutamic-pyruvic transaminase
USG: Ultrasonography
SAAG: Serum ascites albumin gradient
